# Supplementary material for: Exploring the Impact of Workplace Violence in Urban Emergency Departments: A Qualitative Study
Source: Healthcare (Basel). 2025 Mar 20;13(6):679. doi: 10.3390/healthcare13060679 (PMC11941988; doi:10.3390/healthcare13060679)
Supplement: Supplementary file 1 [file healthcare-13-00679-s001.zip › Supplementary/Supplementary S1- UHN Security Operations WPV Quality Improvement Intro and Consent Form.pdf]

UHN Security
PREVENTION • LEADERSHIP • RESPONSE

Introductory Script Template for Measuring, Understanding, Addressing and Ameliorating the Effects Leading to Workplace-Based Violence and Code Whites at UHN Quality Improvement Project

For Surveys and Interview/Focus Group Guides with Staff

Hello and welcome,

You are being invited to take part in an interview for a UHN Quality Improvement (QI) project on workplace-based violence and code whites at UHN emergency departments. This interview is being done to gain an understanding of healthcare providers' current perspectives on workplace-based violence and Code White incidents and feelings of safety and security while working at UHN emergency departments. The information you provide will be used to develop educational materials and UHN security interventions to assist in providing a systematic, comprehensive answer to the problem of emergency department workplace-based violence at UHN.[1]

Taking part in this interview is optional. If you decide not to participate, your employment will not be affected in any way. Information you provide will only be seen by the UHN Security team, consisting of a staff physician, a security director and a research analyst. Others within UHN and outside of UHN will only see a summary of the overall information collected. Your responses will not be linked to your name or personal information in any way and will be stored separately from your personal information. This interview will be recorded and transcribed, the transcription will be kept on the secure UHN server for the two-year period in which this project will be operating. If the results of this interview are published or presented at meetings, your name and other personal identifying information will not be used, and your responses will not be linked to your name or personal information in any way.

If you have questions about this QI project, please contact Christian Schulz-Quach (Christian.schulz-quach@uhn.ca). If you have questions about your rights as a participant in a UHN Quality Improvement Project, please contact theUHN Quality Improvement Review Committee (QIRC) at QI@uhn.ca. QIRC is a group of people who oversee the ethical conduct of QI projects; they are not part of the project team.

Thank you for your participation!

<u>Consent for Future Contact</u> [*<u>Please note</u>: Use this section <u>only</u> if your specific QI project requires follow-up with the same individuals.]*

We are asking for your name and email address to contact you in the future for assessment of the educational materials we develop. If you give us permission to contact you, please fill in your contact information in the space provided. This information will be kept separate from all other information you provide. It will be seen by Dr. Schulz-Quach and kept in an electronic database on a secure UHN server for a five-year period.

Name: ____________________________________

Email/Phone ______________________________

[1] See TCPS 2 (2018) – Chapter 5: Privacy and Confidentiality Section D for a description of consent and secondary use of data, if needed: https://ethics.gc.ca/eng/tcps2-eptc2_2018_chapter5-chapitre5.html
